# Establishment and validation of a prognostic pomogram in unresectable hepatocellular carcinoma treated with intensity modulated radiotherapy: a real world study

**DOI:** 10.1186/s13014-023-02292-7

**Published:** 2023-06-07

**Authors:** Meiying Long, Jianxu Li, Meiling He, Jialin Qiu, Ruijun Zhang, Yingchun Liu, Chunfeng Liang, Haiyan Lu, Yadan Pang, Hongmei Zhou, Hongping Yu, Moqin Qiu

**Affiliations:** 1 Department of Experimental Research, Guangxi Medical University Cancer Hospital, Nanning, 530021 Guangxi China; 2 Department of Epidemiology and Health Statistics, School of Public Health, Guangxi Medical University, Nanning, 530021 Guangxi China; 3 Department of Radiation Oncology, Guangxi Medical University Cancer Hospital, Nanning, 530021 Guangxi China; 4 Oncology Medical College, Guangxi Medical University, Nanning, 530021 Guangxi China; 5 Key Laboratory of Early Prevention and Treatment for Regional High-Frequency Tumor (Guangxi Medical University), Ministry of Education, Nanning, 530021 Guangxi China; 6 Department of Respiratory Oncology, Guangxi Medical University Cancer Hospital, Nanning, 530021 Guangxi China

**Keywords:** Hepatocellular carcinoma, Intensity modulated radiotherapy, Prognostic model, Nomogram, Risk score

## Abstract

**Background:**

To establish a prognostic model to predict the overall survival (OS) in patients with unresectable hepatocellular carcinoma (HCC) treated with intensity modulated radiotherapy (IMRT).

**Methods:**

The unresectable HCC patients treated with IMRT were retrospectively analyzed and randomized into development cohort (n = 237) and validation cohort (n = 103) in a 7:3 ratio. We developed a prognosis model with the multivariate Cox regression analysis in the development cohort to derive the predictive nomogram, which was then validated in the validation cohort. Model performance was evaluated by the c-index, the area under curve(AUC) and the calibration plot.

**Results:**

A total of 340 patients were enrolled. Tumor numbers > 3 (HR = 1.69, 95% CI = 1.21–2.37), AFP ≥ 400 ng/ml (HR = 1.52, 95% CI = 1.10–2.10), PLT < 100 × 10^9(HR = 1.7495% CI = 1.11–2.73), ALP > 150U/L (HR = 1.65, 95% CI = 1.15–2.37) and prior surgery (HR = 0.63, 95% CI = 0.43–0.93) were independent prognostic factors. The nomogram based on independent factors was constructed. The c-index for OS prediction was 0.658 (95% CI, 0.647–0.804) and 0.683 (95% CI, 0.580–0.785) in the development and validation cohort, respectively. The nomogram demonstrated good discriminative ability with AUC rates of 0.726, 0.739 and 0.753 at 1-year, 2-year and 3-year models in the development cohort, and 0.715, 0.756 and 0.780 in the validation cohort, respectively. Additionally, good prognostic discrimination of the nomogram is also reflected in stratifying patients into two subgroups with distinct prognosis.

**Conclusions:**

We constructed a prognostic nomogram for predicting the survival of patients with unresectable HCC treated with IMRT.

**Supplementary Information:**

The online version contains supplementary material available at 10.1186/s13014-023-02292-7.

## Introduction

In 2020, liver cancer ranked as the sixth most common cancer worldwide, with 905,677 new cases [24]. With 830,180 deaths, liver cancer is the third leading cause of cancer-related death worldwide in 2020 [24]. In China, liver cancer ranks fourth in incidence and third in cancer-related mortality among malignancies [6]. Hepatocellular carcinoma (HCC) is the most common primary liver cancer, with a 5-year survival rate of approximately 50% and a recurrence rate of up to 70% [25]. Most HCC patients are diagnosed at an advanced stage and have limited options for systemic therapy, and more than 85% of them are diagnosed with unresectable disease with a poor overall prognosis [12].

Recently, studies on radiotherapy for HCC have reported encouraging results. Of HCC patients with PVTT, radiotherapy significantly improved overall survival (OS) and disease-free survival (DFS) [27]. A retrospective study by Wang et al. [26] investigated the effect of postoperative intensity modulated radiotherapy (IMRT) in 181 HCC patients with a narrow margin (< 1.0 cm) hepatectomy, and found that patients with postoperative IMRT had a better OS and DFS, and fewer early recurrence. For unresectable HCC, radiotherapy combined with TACE is an effective treatment for descending phase transformation [5]. Shui et al. [23] suggested that radiotherapy can be a potential first-line treatment for HCC patients with extensive PVTT originally unsuitable for resection or transarterial chemoembolization (TACE), and the majority of cases achieved tumor thrombus shrinkage and adequate portal vein flow restoration, thus offering them an opportunity to undergo subsequent treatment. At present, as one of the major pillars in the treatment of HCC, radiotherapy has been recommended in the guidelines for the diagnosis and treatment of HCC [8]. However, the diversity of preserved liver function and tumor burden for patients with unresectable HCC explains the large differences in clinical outcomes [4]. Therefore, for patients with unresectable HCC, it is particularly important to establish a set of practical and reliable individualized prognostic assessment systems, which can provide accurate outcome evaluation and obtain more effective therapeutic options. Although prognostic prediction models for radiotherapy have now been developed in several tumor types [16, 21], few studies have focused on the prognosis of patients with unresectable HCC undergoing IMRT.

In this study, we aimed to identify predictors of survival in patients with unresectable HCC and to develop an easily clinically applicable prognostic nomogram to predict OS in unresectable HCC treated with IMRT.

## Materials and methods

### Study patients

The patients in this study were collected from Cancer Hospital. Patients with unresectable HCC who underwent IMRT for liver tumors between March 2012 and December 2021 were enrolled.

The inclusion criteria were: (1) diagnosed with HCC according to histopathology, European Association for the Study of the Liver (EASL) criteria [10] or the American Association for the Study of Liver Diseases Diagnosis (AASLD) [18], (2) received RT plus at least one cycle synchronous, (3) had at least one measurable lesion based on Response Evaluation Criteria in Solid Tumors (RECIST 1.1), (4) Child–Pugh A or B liver function. Patients with intrahepatic cholangiocarcinoma, missing or incomplete clinical data, or follow-up less than 3 months were exempt from the study.

The baseline and tumor characteristics of the patients were available from the electronic medical records. Clinical data are collected on each patient, including their demographics, medical history, liver cancer symptoms, and risk factors. Laboratory data were gathered, including hepatitis B serology, liver function tests, and AFP. In this study, the informed consent of all patients was obtained and approved by Institutional Review Committee of our Cancer Hospital.

### Intensity modulated radiotherapy

All patients underwent enhanced CT scan at 2.5-5 mm slice thickness for IMRT planning. Patients were immobilized by vacuum molds or thermoplastic body films in the supine position with free quiet breathing mode. Gross tumor volume (GTV) was identified as tumor focus. The GTV and organs at risk were contours in the Pinnacle 3 system (Philips, Netherlands) or MIM 6.8 system (MIM, USA). Whenever conditions permitted, CT-positron emission tomography (PET-CT) fusion for the extrahepatic sites and CT-magnetic resonance imaging (MRI) fusion for intrahepatic lesions were performed. For patients with multiple lesions, 1–5 lesions were selected for IMRT at the discretion of the radiation oncologists. IMRT plans were designed using the Monaco treatment planning system (version 5.1) or Pinnacle 3 system (Philips, Netherlands). The final median biologically effective dose used α/β ratio = 10 was 67.2 Gy (IQR, 60–78 Gy). The RT was received using a 6 MV X-ray linear accelerator daily over five fractions per week.

### Outcomes and follow-up

The OS is the outcome of the study, which is calculated from the initiation of IMRT until death or at late follow-up (December 31, 2021). Through the disease management follow-up system of Cancer Hospital, patients are continuously and regularly followed up by professional nurses using active and passive follow-up techniques. The frequency of follow-up was every 3 months for 2 years, every 6 months for 2–5 years, and then, annually after 5 years.

### Statistical analyses

To develop prognostic nomograms and evaluate model predictive performance using a split-sample approach, 340 HCC patients were randomly separated into a training set (n = 237) and a validation set (n = 103) in a 7:3 ratio. Categorical variables were analyzed using the Pearson chi-square test or Fisher exact test, and continuous variables were compared using the Mann–Whitney U test (non-normally distributed data) or the Student's t-test (normally distributed data). Cox proportional hazards models were used to identify prognostic variables, and significant variables (*p* < 0.05) in univariate Cox analysis were included in multivariate analyses. Then, all variables that were statistically significant in the multivariate Cox analysis were used to construct the nomogram, which was performed using R's rms [23] package. The final model was fitted from the stepwise backward selection process based on the Akaike information criterion(AIC). The total area under Receiver Operating Characteristic curve (AUC) was derived to estimate the prediction performance of the risk model. Importance sampling (1,000 bootstraps) was used for the validation of nomogram and calibration curve construction. The risk score model was created by weighting the computed Cox regression coefficients. The risk score for each patient was calculated according to the following formula:

$$Riskscore = \, \sum variable_{i} \times coef_{i}$$

where *variable*_*i*_ is the numerical value(continuous variables) or assigning values(categorical variables) of the ith variable, and *coef*_*i*_ is the coefficient of the ith variable in multivariate Cox regression analysis.

The survival curves were estimated by Kaplan–Meier method and then compared using the log-rank test. A *p* < 0.05 was considered statistically significant. All statistical analyses were performed with R version 4.0.3.

## Results

### Clinical characteristics of patients

A total of 340 patients were included depending on the selection criteria. Among the 340 patients, 90.65% were male. The median patients' age was 55 years, and the most common cause of HCC was chronic HBV infection (74.4%). Based on the histopathological examination, 67.10% of the patients had cirrhotic livers around the hepatic tumor and 56.20% had microvascular invasion (MVI). Among patients who have received prior liver-directed therapy, TACE (64.70%) was the most common preceding treatment. Additionally, some patients received other subsequent treatments after IMRT, including TACE in 58 cases (17.06%), radiofrequency ablation (RFA) in 10 cases (2.94%), surgery in 13 cases (3.82%), and immunotherapy in 37 cases (10.88%). Table 1 shows the baseline characteristics of the two cohorts were no significant differences and were comparable.

**Table 1:** Baseline characteristics of the patients in the development and validation cohort

Variable	Overall cohort (n = 340)	Development cohort (n = 237)	Validation cohort (n = 103)	*p*-value
Age, year	55.11 ± 10.93	55.23 ± 10.92	55.50 ± 10.93	0.757
Sex				
Female	32 (9.4)	21 (8.9)	11(10.7)	0.745
Male	308 (90.6)	216 (91.1)	92 (89.3)	
BMI				
≤ 23 kg/m^2^	251 (73.8)	174 (73.4)	77(74.8)	0.901
> 23 kg/m^2^	89 (26.2)	63 (26.6)	26 (25.2)	
Smoking				
No	170 (50.0)	117 (49.4)	53(51.5)	0.813
Yes	170 (50.0)	120 (50.6)	50 (48.5)	
Drinking				
No	197 (57.9)	138 (58.2)	59(57.3)	0.966
Yes	143 (42.1)	99 (41.8)	44 (42.7)	
Hypertension				
No	294 (86.5)	205 (86.5)	89(86.4)	1.000
Yes	46 (13.5)	32 (13.5)	14 (13.6)	
Diabetes				
No	310 (91.2)	211 (89.0)	99(96.1)	0.056
Yes	30 (8.8)	26 (11.0)	4 (3.9)	
HBsAg				
Negative	82 (25.6)	58(24.5)	29(28.2)	0.562
Positive	253 (74.4)	179 (75.5)	74 (71.8)	
Cirrhosis				
No	112 (32.9)	75 (31.6)	37(35.9)	0.519
Yes	228 (67.1)	162 (68.4)	66 (64.1)	
MVI				
No	149 (43.8)	103 (43.5)	46(44.7)	0.931
Yes	191 (56.2)	134 (56.5)	57 (55.3)	
Extrahepatic metastasis				
No	203 (59.7)	139 (58.6)	64(62.1)	0.630
Yes	137 (40.3)	98 (41.4)	39 (37.9)	
Tumor size				
≤ 6 cm	172 (50.6)	118 (49.8)	54(52.4)	0.742
> 6 cm	168 (49.4)	119 (50.2)	49 (47.6)	
Tumors number				
≤ 3	180 (52.9)	123 (51.9)	57(55.3)	0.641
> 3	160 (47.1)	114 (48.1)	46 (44.7)	
BCLC stage C				
No	72 (21.2)	49 (20.7)	23(22.3)	0.842
Yes	268 (78.8)	188 (79.3)	80 (77.7)	
Child Paugh class				
A	259 (76.2)	188 (79.3)	71(68.9)	0.054
B	81 (23.8)	49 (20.7)	32 (31.1)	
BED				
≤ 60 Gy	123 (36.2)	87 (36.7)	36(35.0)	0.852
> 60 Gy	217 (63.8)	150 (63.3)	67 (65.0)	
PLT, (× 10^9^/L)				
≥ 100	294 (86.5)	204(86.1)	90(87.4)	0.881
< 100	46 (13.5)	33 (13.9)	13 (12.6)	
ALC, (× 10^9^/L)				
≤ 1.8	277 (81.5)	194 (81.9)	83(80.5)	0.900
> 1.8	63 (18.5)	43 (18.1)	20 (19.4)	
ANC, (× 10^9^/L)				
< 2	307 (90.3)	217 (91.6)	90(87.3)	0.318
≥ 2	33 (9.7)	20 (8.4)	13 (12.6)	
Hb				
≥ 131 g/L	147 (43.2)	102 (43.0)	45(44.7)	1.000
< 131 g/L	193 (56.8)	135(57.0)	58 (56.3)	
AST				
≤ 40 U/L	163 (47.9)	116 (48.9)	47(45.6)	0.657
> 40 U/L	177 (52.1)	121 (51.1)	56 (54.4)	
ALT				
≤ 40 U/L	199 (58.5)	137 (57.8)	62(60.2)	0.771
> 40 U/L	141 (41.5)	100 (42.2)	41 (39.8)	
ALP				
≤ 150 U/L	241 (70.9)	168 (70.9)	73(70.9)	1.000
> 150 U/L	99 (29.1)	69 (29.1)	30 (29.1)	
TBIL				
≤ 21 umol/L	257 (75.6)	177 (74.7)	80(77.7)	0.651
> 21 umol/L	83 (24.4)	60 (25.3)	23 (22.3)	
Alb				
≥ 55 g/L	188 (55.3)	132 (55.7)	56(54.4)	0.914
< 55 g/L	152 (44.7)	105 (44.3)	47 (45.6)	
PT				
≤ 13 s	205 (60.3)	147 (62.0)	58(56.3)	0.385
> 13 s	135 (39.7)	90 (38.0)	45 (43.7)	
AFP				
< 400 ng/ml	218 (64.1)	150 (63.3)	68(66.0)	0.720
≥ 400 ng/ml	122 (35.9)	87 (36.7)	35 (34.0)	
Prior TACE				
No	120 (35.3)	80 (33.8)	40(38.8)	0.437
Yes	220 (64.7)	157 (66.2)	63 (61.2)	
Prior surgery				
No	170 (50.0)	124 (52.3)	46(44.7)	0.238
Yes	170 (50.0)	113 (47.7)	57 (55.3)	
Prior immunotherapy				
No	328 (96.5)	229 (96.6)	99(96.1)	1.000
Yes	12 (3.5)	8 (3.4)	4 (3.9)	
Combined immunotherapy				
No	311 (91.5)	214 (90.3)	97(94.1)	0.334
Yes	29 (8.5)	23 (9.7)	6 (5.8)	

*AFP* alpha-fetoprotein; *BCLC* barcelona clinic liver cancer; *BMI* body mass index; *MVI* macrovascular invasion; *BED* biologically effective dose; *TACE* transarterial chemoembolization; *PLT* platelet; *ALC* Absolute Lymphocyte Count; *ANC* Absolute Neutrophil Count; *Hb* Hemoglobin; *AST* Aspartate aminotransferase; *ALT* Alanine aminotransferase; *ALP* alkaline phosphatase; *TBIL* total bilirubin; *Alb* Albumin; *PT* Prothrombin time

During a median follow-up of 24.7 months (range, 1.53 to 83.73 months), 243 deaths occurred and 97 patients were alive at the last follow-up. The median OS of the primary cohort was 12.1 months (Interquartile range, 8.57–24.71) with survival rates of 63.66%, 25.11%, and 13.78% at 1, 2, and 3 years, respectively. The 1-year, 3-year and 5-year OS rates were 77.4%, 52.5%, and 25.2% for the training cohort, and 76.7%, 53.0%, and 24.5% for the validation cohort, respectively. The survival curve did not differ considerably between the development cohort and the validation cohort (Additional file 1: Fig. S1).

### Development of the models from the development cohort

In univariate analysis, 12 factors (extrahepatic metastasis, tumor size, tumor number, BCLC stage, Child-Paugh class, PLT, ALC, ALP, Alb, AFP, and prior surgery) were considered to be significant survival-related variables among the 33 candidate variables in the training cohort (Table 2) and further included in multivariate analysis. Multivariate analysis identified that tumor numbers > 3, AFP ≥ 400 ng/ml, PLT < 100 × 10^9 ng/ml, and ALP > 150U/L were independent risk factors of OS, while prior surgery was an independent protective factor (Table 2).

**Table 2:** Univariable and multivariable analysis for factors affecting overall survival

Variable	Univariable analysis		Multivariable analysis	
	HR (95% CI)	*p*-value	HR (95% CI)	*p*-value
Age	1.00 (0.99–1.02)	0.869		
Sex (male vs. female)	1.35 (0.78–2.34)	0.284		
BMI (> 23 kg/m^2^ vs. ≤ 23 kg/m^2^)	0.88 (0.62–1.23)	0.445		
Smoking (yes vs. no)	1.03 (0.77–1.4)	0.823		
Drinking (yes vs. no)	0.96 (0.71–1.31)	0.810		
Hypertension (yes vs. no)	0.92 (0.59–1.44)	0.717		
Diabetes (yes vs. no)	1.39 (0.88–2.2)	0.156		
HBsAg (positive vs. negative)	1.16 (0.82–1.66)	0.399		
Cirrhosis (yes vs. no)	1.29 (0.93–1.8)	0.129		
MVI (yes vs. no)	1.29 (0.95–1.75)	0.097		
Extrahepatic metastasis (yes vs. no)	1.36 (1.01–1.84)	0.046	1.27 (0.88–1.83)	0.198
Tumor size (> 6 cm vs. ≤ 6 cm)	1.7 (1.26–2.31)	0.001	0.86 (0.58–1.28)	0.465
Tumor number (> 3 vs. ≤ 3)	2.13 (1.57–2.89)	< 0.001	1.69 (1.21–2.37)	0.002
BCLC stage C (yes vs. no)	1.54 (1.05–2.25)	0.027	1.09 (0.70–1.72)	0.697
Child Paugh class (B vs. A)	1.47 (1.03–2.12)	0.036	0.94 (0.61–1.45)	0.770
BED (> 60 Gy vs. ≤ 60 Gy)	0.80 (0.59–1.09)	0.161		
PLT (< 100 × 10^9^/L vs. ≥ 100 × 10^9^/L)	1.82 (1.19–2.79)	0.006	1.74 (1.11–2.73)	0.017
ALC (> 1.8 × 10^9^/L vs. ≤ 1.8 × 10^9^/L)	0.58 (0.39–0.88)	0.010	0.78 (0.50–1.20)	0.260
ANC (≥ 2 × 10^9^/L vs. < 2 × 10^9^/L)	1.54 (0.92–2.59)	0.101		
Hb (< 131 g/L vs. ≥ 131 g/L)	1.50 (1.11–2.03)	0.009	1.31 (0.92–1.84)	0.131
AST (> 40 U/L vs. ≤ 40 U/L)	1.26 (0.94–1.71)	0.126		
ALT (> 40 U/L vs. ≤ 40 U/L)	1.03 (0.76–1.4)	0.839		
ALP (> 150 U/L vs. ≤ 150 U/L)	1.80 (1.31–2.48)	< 0.001	1.65 (1.15–2.37)	0.006
TBIL (> 21umol/L vs. ≤ 21umol/L)	1.36 (0.97–1.92)	0.076		
Alb (< 55 g/L vs. ≥ 55 g/L)	1.43 (1.06–1.93)	0.021	1.06 (0.73–1.56)	0.746
PT (> 13 s vs. ≤ 13 s)	1.21 (0.89–1.65)	0.213		
AFP (≥ 400 ng/ml vs. < 400 ng/ml)	1.45 (1.07–1.98)	0.017	1.52 (1.10–2.10)	0.011
Prior TACE (yes vs. no)	1.04 (0.75–1.43)	0.817		
Prior surgery (yes vs. no)	0.53 (0.39–0.72)	< 0.001	0.63 (0.43–0.93)	0.020
Prior immunotherapy (yes vs. no)	1.89 (0.77–4.63)	0.165		
Combined immunotherapy (yes vs. no)	1.15 (0.65–2.05)	0.627		

*AFP* alpha-fetoprotein; *BCLC* Barcelona Clinic Liver Cancer; *BMI* Body Mass Index; *MVI* macrovascular invasion; *BED* biologically effective dose; *TACE* transarterial chemoembolization; *PLT* platelet; *ALC* Absolute Lymphocyte Count; *ANC* Absolute Neutrophil Count; *Hb* Hemoglobin; *AST* Aspartate aminotransferase; *ALT* Alanine aminotransferase; *ALP* alkaline phosphatase; *TBIL* total bilirubin; *Alb* Albumin; *PT* Prothrombin time; *HR* hazard ratio; *CI* confidence interval

**Fig. 1:**
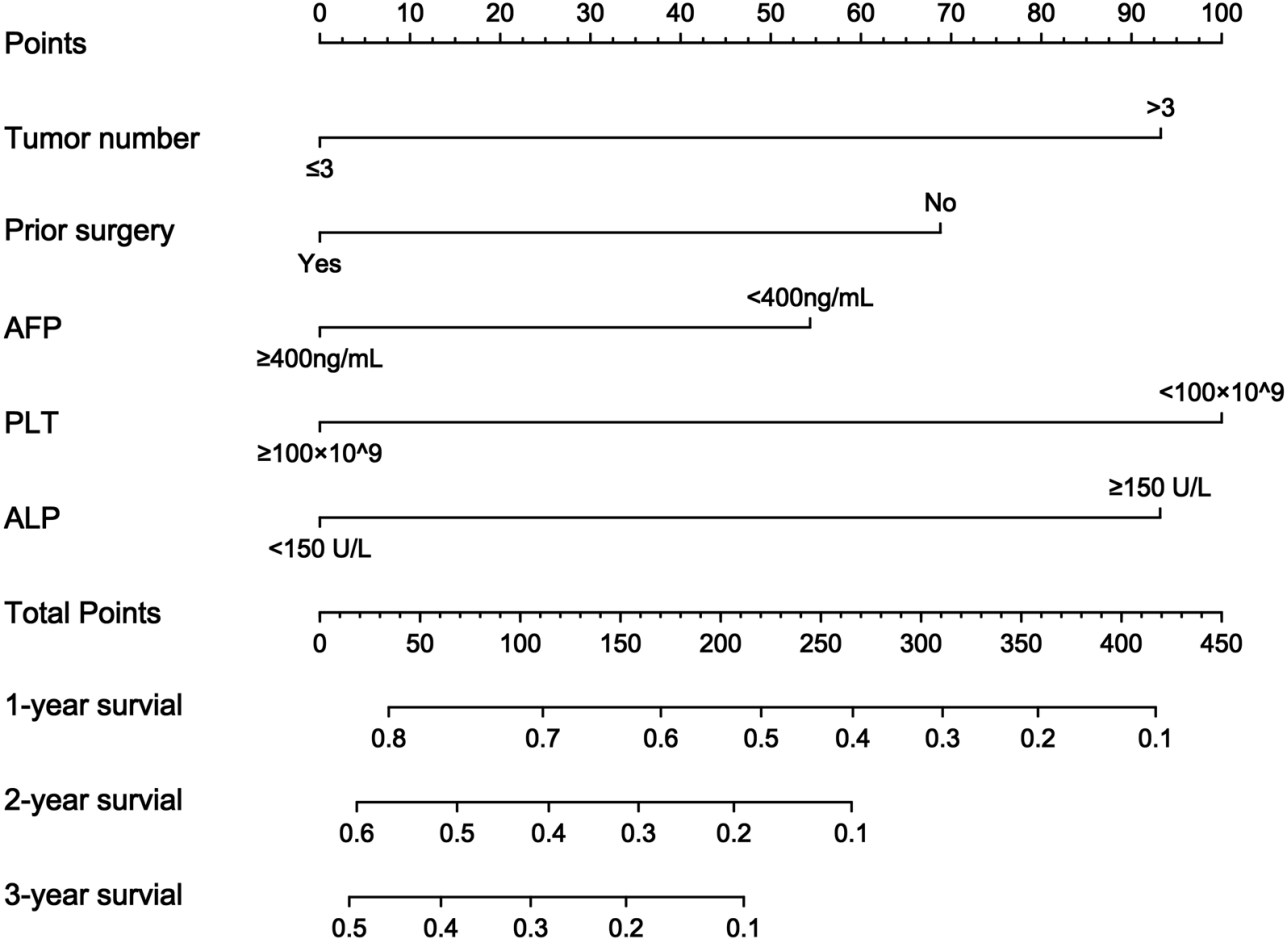
Prognostic nomogram estimated by clinical features. The total points of each patient can be used to predict the overall 1-year, 2-year and 3-year survival rates in unresectable HCC patients after IMRT. HCC, hepatocellular carcinoma; IMRT, intensity modulated radiotherapy

Figure 1 displays the Nomograms incorporating all the aforementioned significant independent predictors based on the multivariate Cox proportional hazards model. The nomogram illustrated tumor number as sharing the largest contribution to the OS with the regression coefficients of 0.45 in the Cox model, while prior surgery, AFP, PLT, and ALP showed a moderate impact on the OS. Each category within these variables was assigned a score on the point scale.

### Predictive performance of the nomogram to predict overall survival in the development cohort

**Fig. 2:**
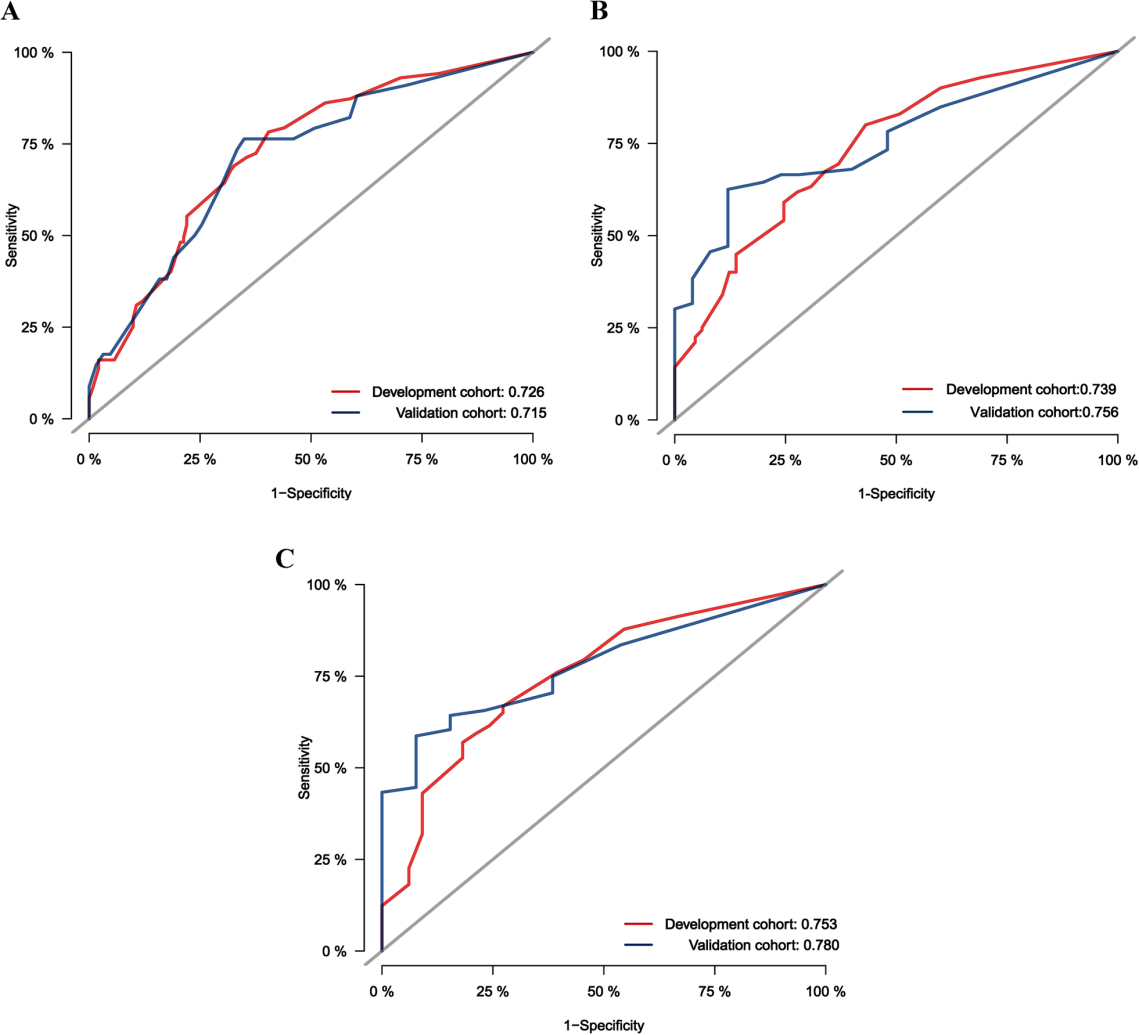
ROC curves and AUC for the prediction of death within 1 year (**A**), 2 years (**B**) and 3 years (**C**) among HCC patients after IMRT in the development and validation cohort. ROC, receiver operating characteristic; AUC, area under the curve; HCC, hepatocellular carcinoma, IMRT, intensity modulated radiotherapy

**Fig. 3:**
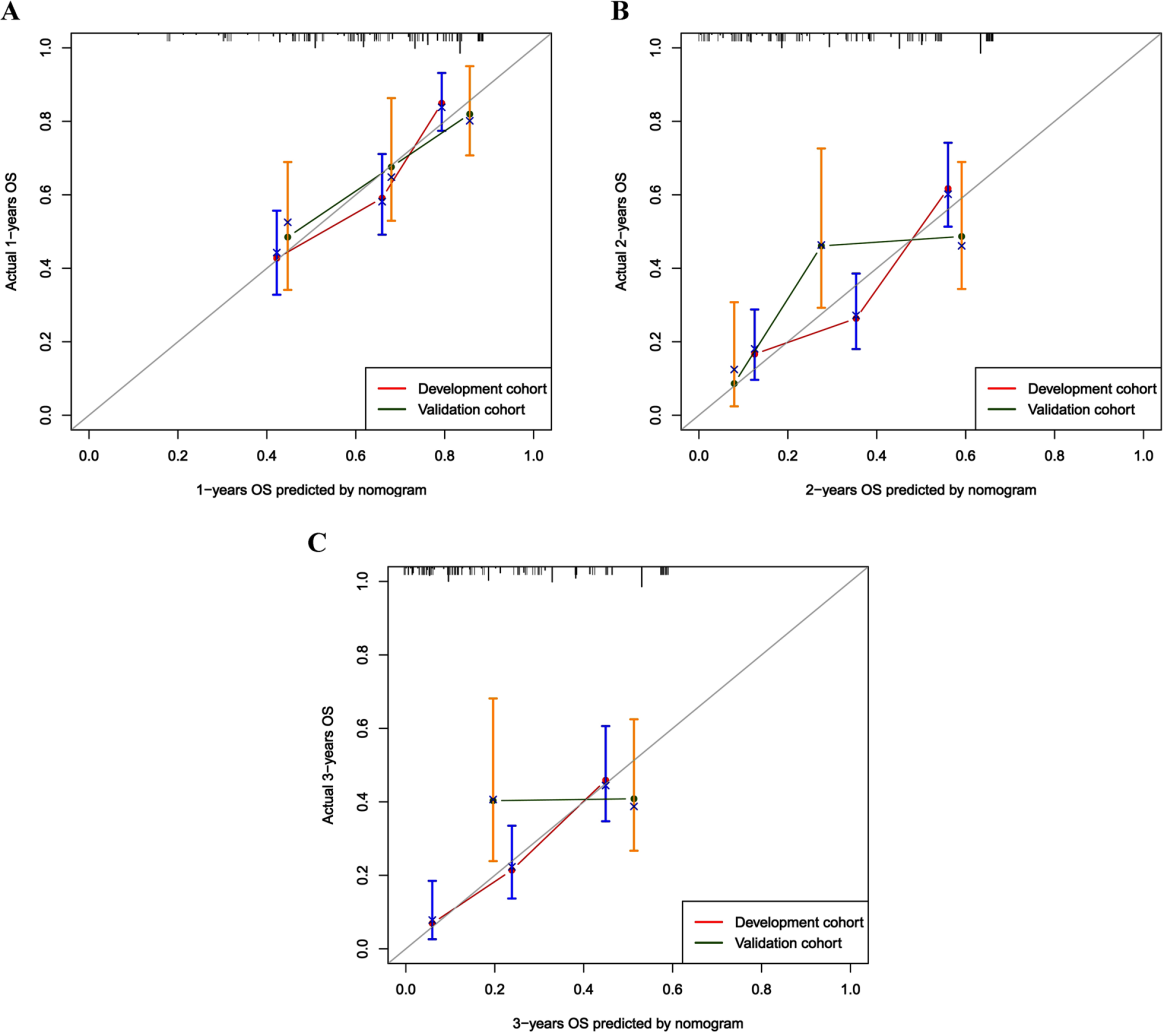
Calibration curve for the prediction of death within 1-year (**A**), 2-year (**B**) and 3-year (**C**) among HCC patients after IMRT in the development and validation cohort. HCC, hepatocellular carcinoma, IMRT, intensity modulated radiotherapy

In the development cohort, the c-index for OS prediction were 0.658 (95% CI, 0.647–0.804), indicating good performance in OS prediction. The development cohort showed an AUC of 0.739(95% CI, 0.668–0.811), 0.726(95% CI, 0.660–0.793), and 0.753(95% CI, 0.664–0.842) for 1-year, 2-year, and 3-year, respectively (Fig. 2A–C). The calibration plot for the probability of survival showed an optimal agreement between prediction by the nomogram and the actual observation (Fig. 3A–C).

### Internal validation of predictive accuracy of the nomogram for overall survival

Across the internal validation cohort, the nomogram also had good performance in OS prediction, with C-indexes of 0.683 (95% CI, 0.580–0.785). According to Fig. 2, the validation cohort's AUC for the 1-, 2-, and 3-year was 0.715(95% CI, 0.608–0.822), 0.756(95% CI, 0.653–0.859), and 0.780(95% CI, 0.668–0.891), respectively (Fig. 2A–C). The calibration curves demonstrated good corresponds to the predicted and observed probability, with a goodness-of-fit of Hosmer–Lemeshow test (X^2^ = 5.210, *p* = 0.634) (Fig. 3A–C).

### Discriminatory power of the nomogram

**Fig. 4:**
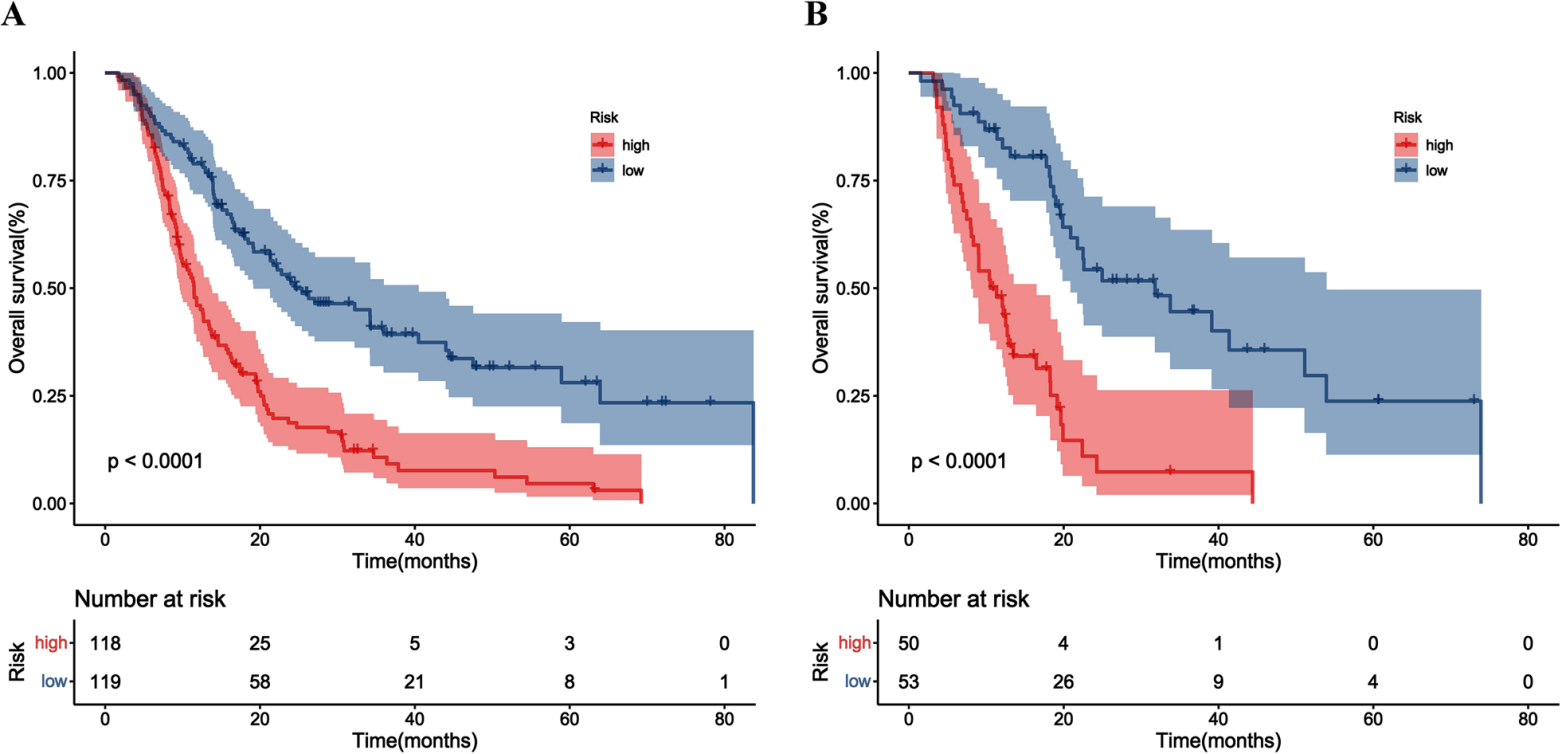
Survival curves of patients with high-risk and low-risk: development cohort (**A**) and validation cohort (**B**)

To evaluate the discriminatory ability of the prognostic nomogram, patients were classified into two subgroups (high-risk, and low-risk stages) according to a median risk score. Kaplan–Meier curves are shown that the model could also accurately stratify patients into clearly different prognostic subgroups (*p* < 0.001) (Fig. 4). The 3-year OS rates for the high- and low-risk groups were 7.6% and 37.4% in the development cohort, and 0% and 40.1% in the validation cohort, respectively.

## Discussion

IMRT has been shown to provide a survival benefit in certain patients with unresectable HCC [9], therefore, it is important to stratify patients for the potential benefit of IMRT. We developed and validated a nomogram for predicting the probability of survival at 1, 2, and 3 years in patients with unresectable HCC after treatment with IMRT. Currently, the nomogram now significantly integrates clinicopathologic prognostic risk factors and treatment means. The nomogram also performs well in predicting survival based on the results of the AUC and calibration curves. Patients were split by the nomograms into different prognostic subgroups to provide individualized OS risk estimates.

The survival rate of unresectable HCC patients treated with IMRT is encouraging when compared to other treatment modalities. Despite advances in target therapy in different subgroups of HCC patients, the survival rate of patients remains poor, as well as emerging immune checkpoint inhibitors [30]. Standard therapies for unresectable HCC are currently nonexistent. Several complementary nomograms based on different treatment modalities have been proposed by scholars. Xu et al. developed a nomogram to predict survival for unresectable HCC with TACE-treated, consisting of vascular invasion, tumor number, preserved the tumor capsule, AFP levels, AST levels, and indocyanine green retention rate [28]. This nomogram outperformed seven frequently used staging methods in terms of prediction accuracy and discriminating ability. Huang et al. derived a prognostic model based on tumor size, tumor number, microvascular invasion, CTP liver function grade, and biologically effective dose that was a good predictor of prognosis for non-metastatic advanced HCC patients who received SBRT [13]. Besides, predictive nomograms are also available in radiofrequency ablation [14] and surgical treatment [29].

In the present nomogram, independent prognostic factors for tumor number, previous surgical resection, AFP level, PLT level, and ALP level were included. Tumor number was identified as an independent risk factor for OS in this study, which once again demonstrated that unresectable HCC with a high intrahepatic disease burden shows a poorer prognosis after IMRT. Hepatic resection is the first treatment choice for most patients with HCC, although less than 30% of patients are potentially eligible for treatment [22]. However, the survival rate after resection is unsatisfactory, and most patients experienced relapse after resection, with a 5-year post-operative recurrence of 60–70% [11]. In this study, patients who received IMRT treatment are no longer suitable or intolerant of first-line treatment, and 50% of them have a history of surgical resection. Our analysis shows that surgical treatment before IMRT is a protective factor for unresectable HCC and a key component of the nomogram. The reason behind this phenomenon potentially is that the intrahepatic tumor burden of patients after resection has been alleviated to a certain extent [1], and their VMI status has been improved [7]. Given the particular role of surgical history, a unique nomogram will need to be constructed in the future to better explore this subset of patients.

The nomogram also included three semi-synthetic measures of AFP, PLT, and ALP, and elevated them were involved a significantly poorer OS. AFP has been recognized as one of the most important prognostic variables in HCC, which is incorporated into HCC staging systems of CUPI and GERTCH [2]. Although there is no evidence to support an absolute cutoff value for AFP in prognosis, it has been shown that AFP level ≥ 400 ng/ml contributed more to the prognostic nomogram of unresectable HCC in a large multicenter study [28], which is consistent with our research. Morimoto et al. discovered, through a retrospective study of 1613 HCC patients, that higher peripheral platelet count was an independent risk factor for extrahepatic metastasis [19]. Basic studies have confirmed that PLT can avoid natural killer cells to induce immune escape through the mediation of platelet glycoprotein (GP) II b/III a and P-selectin, consequently improving the survival rate of HCC tumor cells and promoting the development of tumor thrombus [15]. Meanwhile, elevated serum ALP levels always occur in liver disease that may reflect liver damage [20]. Moreover, research indicates that high pretreatment serum ALP levels are considered to strongly correlate with poor survival in HCC patients [17]. By applying the model's risk score, we were able to separate patients into two subgroups with significantly different clinical outcomes, suggesting that our nomogram is a valid tool for predicting survival in unresectable HCC patients undergoing IMRT. A comprehensive assessment based on our nomogram information will assist physicians or(and) patients in choosing the best treatment options, especially for those who may take advantage of receiving IMRT, otherwise an alternative treatment choice [3, 11].

Several limitations of our investigation must be acknowledged. First, this is a retrospective, a single-center, historical cohort study with inherent selection and referral bias constraints. Consequently, the generalizability of our findings will be restricted, and multicenter, prospective studies are necessary for further validation. Second, the applicability of our nomogram to patients from other countries and/or with various etiologies remains unverified, given that the majority of our research participants were HBV-related HCC and from Southern China. In addition, despite the widespread use of IMRT, the number of HCC patients treated by this method in our hospital is modest, and the sample size of our study is relatively small.

In conclusion, we developed a nomogram to predict survival in unresectable HCC patients treated with IMRT. The nomogram facilitates individualized prognosis prediction of unresectable HCC patients treated with IMRT and provides recommendations for pre-treatment decision-making by physicians and/or patients. Larger, multi-institutional patient cohorts are required to validate the model's reliability and validity.

## Supplementary Information

**Additional file 1**. **Fig. S1**. The survival curve between the development cohort and the validation cohort.

## Data Availability

Not applicable.

## Abbreviations

HCC: Hepatocellular carcinoma
IMRT: Intensity modulated radiotherapy
OS: Overall survival
BCLC: Barcelona clinic liver cancer
BMI: Body mass index
MVI: Macrovascular invasion
BED: Biologically effective dose
TACE: Transarterial chemoembolization
HR: Hazard ratio
CI: Confidence interval
ROC: Receiver operating characteristic
AUC: Area under the curve
