# Supplementary figures and images for: Establishment and validation of a prognostic pomogram in unresectable hepatocellular carcinoma treated with intensity modulated radiotherapy: a real world study
Source: Radiat Oncol. 2023 Jun 7;18:96. doi: 10.1186/s13014-023-02292-7 (PMC10245442; doi:10.1186/s13014-023-02292-7)

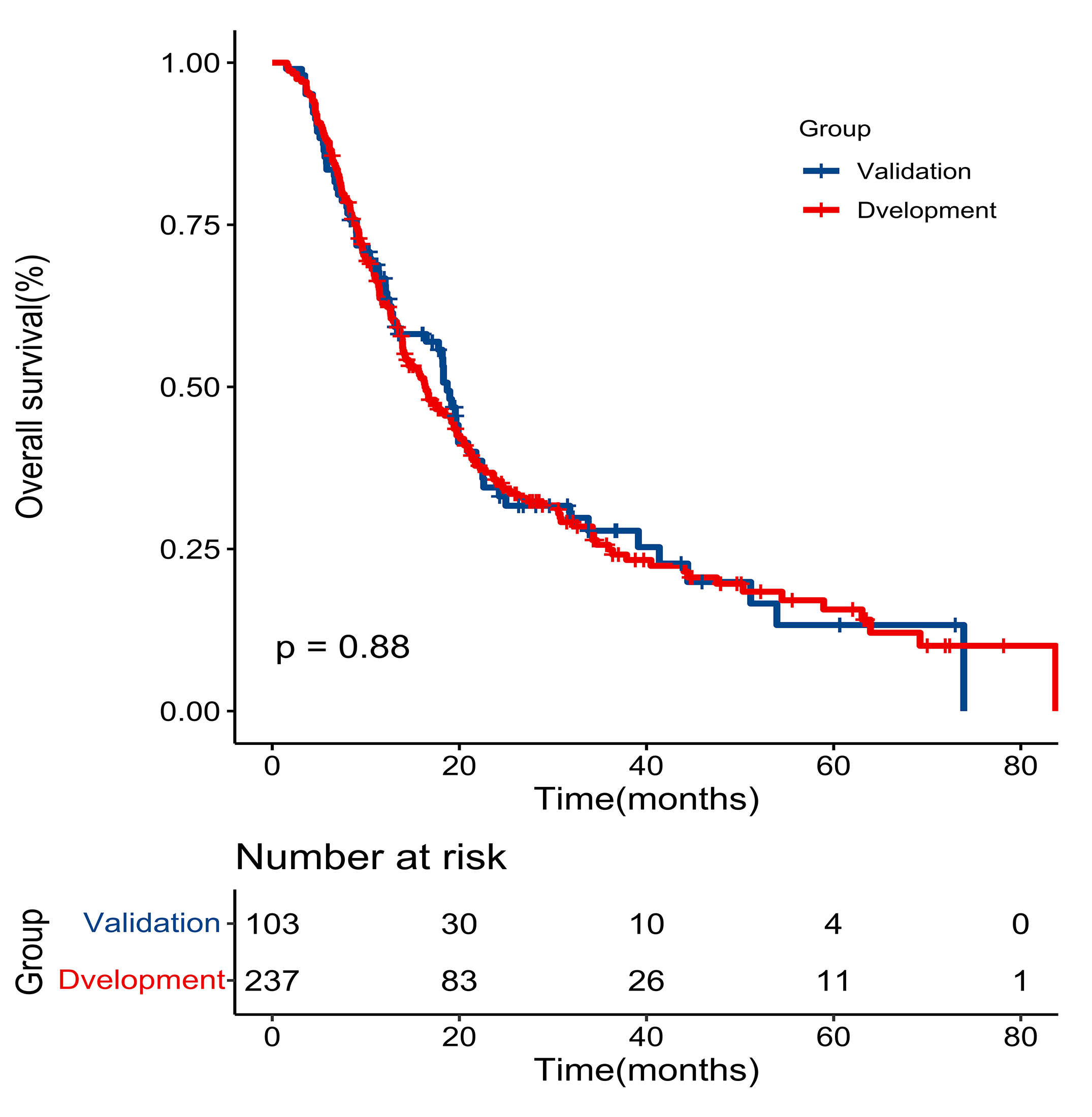

Supplement: Supplementary file 1 — Additional file 1. Fig. S1. The survival curve between the development cohort and the validation cohort. [file 13014_2023_2292_MOESM1_ESM.tif]
